# Data‐Driven Identification of Rational Nonlinear Dynamics in Biochemical Networks via an Implicit Singular Value Decomposition Based Framework

**DOI:** 10.1049/syb2.70068

**Published:** 2026-05-14

**Authors:** Hongtao Zhu, Longwei Zuo, Chunjian Pan, Qingchao Jiang, Zhixing Cao

**Affiliations:** ^1^ Key Laboratory of Smart Manufacturing in Energy Chemical Process Ministry of Education East China University of Science and Technology Shanghai China; ^2^ College of Automation Engineering Shanghai University of Electric Power Shanghai China; ^3^ Department of Chemical Engineering Queen's University Kingston Ontario Canada

**Keywords:** biochemistry, biology computing, data analysis, nonlinear dynamical systems, singular value decomposition

## Abstract

Using rational functions to describe the dynamics of the biological network is common in the field of biology. Estimating such dynamics from data is challenging owing to the complex nonlinearities. Although sparse identification of nonlinear dynamics (SINDy) approach works for many systems, it struggles with rational functions because constructing separate basis libraries for numerators and denominators is computationally prohibitive. Hence, this study proposes an effective method to identify the rational form dynamics of complex biological networks. The method applies singular value decomposition (SVD) to a library of observational functions that mix state and derivative terms, capturing the implicit form of the rational functions. The null space obtained from this SVD analysis is then used for model identification. The data information was condensed into the observable space through the SVD approach. The computational cost to obtain the null space depends on the size of the observable space. Therefore, it can easily handle large‐scale data. The proposed method was successfully applied to four biological models: (1) Michaelis–Menten kinetics, (2) *Bacillus subtilis* competence network, (3) penicillin production kinetics and (4) the yeast glycolytic metabolic network, demonstrating its effectiveness and generalisability across different biological systems.

## Graphical abstract

This study introduces a novel method to identify rational‐function dynamics in biological networks, overcoming the limitations of existing approaches like SINDy. By applying singular value decomposition to a mixed library of state and derivative terms, the method efficiently extracts the implicit model structure from data. Its effectiveness is demonstrated by the successful application to four diverse biological systems, including metabolic and regulatory networks.

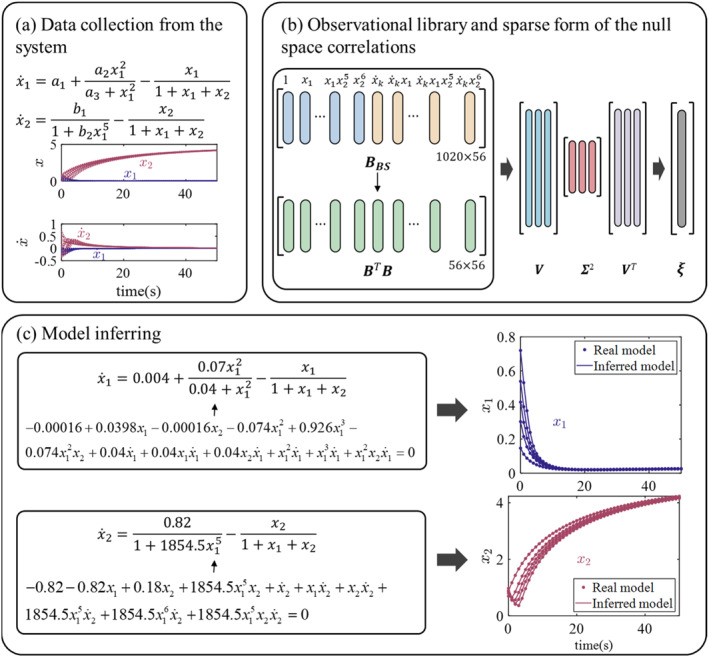

## 1 Introduction

The escalating complexity of biological systems has made the precise characterisation of metabolic and regulatory network dynamics a pivotal challenge in advancing biological research [1, 2]. Contemporary biological networks frequently exhibit highly nonlinear dynamical behaviours, necessitating sophisticated modelling approaches for accurate representation—a requirement particularly critical in metabolic regulation studies because of their direct implications for disease mechanism elucidation and therapeutic development [3]. Recent breakthroughs in high‐throughput sequencing [4] and microarray technologies [5] have provided unprecedented data support for revealing multilayered molecular interaction networks. However, the inherent requirement for rational function representations in modelling such system dynamics continues to pose significant challenges for both structural identification and parameter estimation. This context underscores the crucial need to develop data‐driven methodologies capable of efficiently identifying and reconstructing rational function‐based dynamical models [6, 7, 8, 9]. Such approaches not only facilitate the elucidation of topological structures and interaction mechanisms within biological networks but also offer novel theoretical perspectives for understanding their functional complexity.

Traditional methods for inferring the dynamics of biological networks primarily include graph‐based models, differential equation‐based approaches and machine learning techniques. Although these methods are powerful tools for biological network analysis, they face specific challenges and limitations. Graph‐based models such as Boolean networks [10, 11, 12] excel at managing large‐scale networks and conducting qualitative analyses. However, they often struggle to capture quantitative dynamics and are sensitive to parameter variations. Differential equation‐based methods [13, 14, 15, 16] can deliver detailed insights into dynamic changes but require extensive parameter estimation and can be complex to solve, particularly for nonlinear systems. Machine learning methods [17, 18, 19, 20] effectively handle high‐dimensional data but are constrained by data quantity and quality, often lacking interpretability.

Recent approaches have focused on automating model discovery through techniques such as Bayesian inference and sparse regression, which enhance the interpretability of inferred models. Bayesian methods [21, 22, 23, 24] are widely used for inferring nonlinear dynamics because of their ability to incorporate prior knowledge and uncertainty into the modelling process, enabling robust inference even with incomplete or noisy data. However, Bayesian methods are often computationally expensive owing to the need for high‐dimensional data processing, complex numerical sampling techniques and solving high‐dimensional integrals to account for sparse network structures and stable dynamics. The sparse identification of nonlinear dynamics (SINDy) algorithm provides a powerful framework for modelling complex dynamical systems [25, 26, 27, 28, 29, 30]. To address the challenge of estimating rational dynamics directly using SINDy, implicit‐SINDy [31] has successfully inferred canonical biological models with rational function nonlinearities. SINDy‐PI [32] further extends this framework by introducing a parallel implicit identification approach that significantly improves robustness to noise, enabling the discovery of implicit and rational dynamics in previously inaccessible systems. However, both implicit‐SINDy and SINDy‐PI require extensive iterations to reach sparse solutions, which makes them computationally expensive, especially when dealing with complex rational dynamics. This is due to the fact that identifying the sparsest solution within the null space is inherently a nonconvex optimisation problem. Additionally, noise in the data affects the rank of the feature library matrix and alters the dimension and structure of the null space, thereby presenting significant challenges for model identification.

This paper introduces an algorithm based on singular value decomposition (SVD) analysis on an observable matrix to estimate dynamic models from data. The observable matrix is obtained by reducing the dimensionality of the observational library. Through the SVD analysis of the observable matrix, the information in the data is condensed into the observable space to capture the implicit form of rational functions of a biological system. Hence the proposed approach is computationally efficient for large‐scale data problems, as its computational cost depends on the size of the observable matrix (i.e., the number of the basis functions). Then, SVD is employed to obtain the null space of the observable matrix, thus eliminating the need for sparse optimisation [33] to determine the solution [34]. SVD‐based null‐space computation typically uses bidiagonalisation, which reduces the computational complexity by simplifying the matrix and helps extract a robust null space representation [35]. The proposed method is evaluated on four biological network models: Michaelis–Menten kinetics, the regulatory network for competence in *Bacillus subtilis*, penicillin production kinetics and the yeast glycolytic metabolic network.

The contents of the paper are organised as follows. After the introduction, Section 2 provides a theoretical description of the proposed method. In Section 3, the algorithm is tested on four significant biological network examples compared to the performance of implicit‐SINDy. Finally, we present conclusions regarding the effectiveness of the proposed approach.

## 2 Inferring Rational Form Dynamics Based on SVD

### 2.1 Observational Library Construction for Rational Dynamic Functions

**FIGURE 1:**
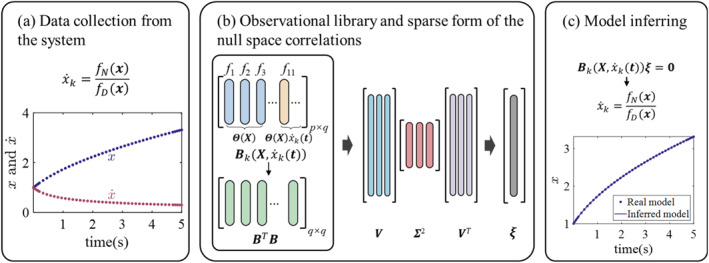
The framework of the proposed approach. Data are initially collected from the system dynamics and then used to construct an observational library. Subsequently, the observational library is dimensionally reduced to form the observable matrix. SVD is applied to determine the null space thus identifying correct active terms. Finally, the system dynamics equations are reconstructed based on these derived components.

This paper specifically focuses on systems with nonlinear rational terms as follows:

(1) $${\dot{x}}_{k}=\frac{N\left(x\right)}{D\left(x\right)}$$

where $x={\left[x_{1},x_{2},\ldots ,x_{n}\right]}^{T}$ represents the system state variables, k takes values ranging from 1 to n (indicating the number of state variables) and ${\dot{x}}_{k}$ denotes the derivative of the k‐th state variable. The terms N(x) and D(x) represent the numerator and denominator polynomials, respectively. This structure mirrors classical biological network models, capturing the complex relationships between reaction rates and substance concentrations. The proposed procedure for identifying the rational form dynamics is illustrated in Figure 1.

First, the time‐series data matrix is sourced from the system (assuming Equation (1) is the rational function model to be identified):

(2) $$X={\left[\begin{array}{cccc} x\left(t_{1}\right) & x\left(t_{2}\right) & ... & x\left(t_{p}\right) \end{array}\right]}^{T}$$

where $x\in R^{n}$, *p* is the number of time points and [⋯]^*T*^ represents the matrix transpose; $\dot{X}={\left[\dot{x}\left(t_{1}\right)\;\dot{x}\left(t_{1}\right)\ldots \;\dot{x}\left(t_{p}\right)\right]}^{T}$ is the time derivative matrix.

Since N(x) and D(x) are both polynomials, the polynomial library Θ(X) can be constructed as follows:

(3) $$\Theta \left(X\right)=\left[\begin{array}{cccccccc} 1 & X & X^{2} & ... & X^{d} & ... & \sin\left(X\right) & ... \end{array}\right]$$

where $X^{d}$ denotes the matrix containing all possible column vectors obtained from time‐series of the d‐th degree polynomials in the state vector x. For example, for a system with two states $x={\left[x_{1},x_{2}\right]}^{T}$, the matrix $X^{2}=\left[x_{1}^{2}\left(t\right),\left(x_{1}x_{2}\right)\left(t\right),x_{2}^{2}\left(t\right)\right]$, where ***t*** is a vector of times at which the state is measured.

Then, the observational library $B\left(X,{\dot{x}}_{k}\left(t\right)\right)$ for the rational equation of ${\dot{x}}_{k}\left(t\right)$ is constructed by including both the state variables x and its time derivative ${\dot{x}}_{k}$

(4) $$B\left(X,{\dot{x}}_{k}\left(t\right)\right)=\left[\Theta \left(X\right)\;{\dot{x}}_{k}\left(t\right)\Theta \left(X\right)\right]$$

where $B\left(X,{\dot{x}}_{k}\left(t\right)\right)\in R^{p\times q}$ (*q* is the size of the observational library), and the second term ${\dot{x}}_{k}\left(t\right)\Theta \left(X\right)$ represents the product of the polynomial library and the state time derivative ${\dot{x}}_{k}\left(t\right)$. The observational library captures all relevant terms up to degree *d*, accurately reflecting the nonlinear dynamics of the system by including both linear and higher‐order interactions.

### 2.2 Sparse Form of the Null Space Correlations by SVD

There is an implicit problem form of Equation (1):

(5) $${\dot{x}}_{k}D\left(x\right)=N\left(x\right)$$

Considering such a form and observational library, the implicit problem statement can be reformulated as follows:

(6) $${\dot{x}}_{k}D\left(x\right)-N\left(x\right)=0\to B\left(X,{\dot{x}}_{k}\left(t\right)\right)\xi$$

**FIGURE 2:**
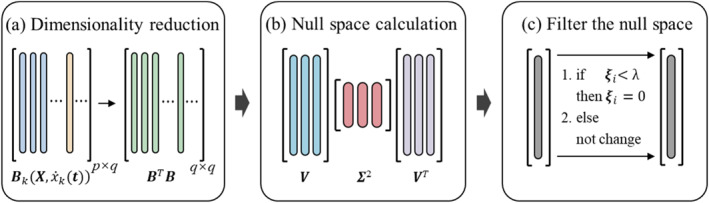
Algorithm to extract the sparse form of the null space.

The key step of the algorithm is to extract the sparse form ξ of the null space (illustrated in Figure 2).

The SVD of B can be expressed as follows:

(7) $$B=U\Sigma V^{T}$$

where U is the matrix of left singular vectors, Σ is the matrix of singular values and V is the matrix of right singular vectors. The column vector of the right singular vector matrix corresponding to the singular value closest to zero is the null space of the observational library.

Therefore, the observable matrix can be expressed as follows:

(8) $$B^{T}B=V\Sigma^{T}U^{T}U\Sigma V^{T}$$

and U is an orthogonal matrix, (i.e., $U^{T}U=I$, $\Sigma^{T}\Sigma =\Sigma^{2})$, and the following equation holds true:

(9) $$B^{T}B=V\Sigma^{2}V^{T}$$

By comparing Equation (9) with Equation (7), it is evident that the right singular vectors of matrix B correspond to the eigenvectors of $B^{T}B$. In other words, ***V*** can be obtained by applying SVD to $B^{T}B$. Meanwhile, the observable matrix achieves dimensionality reduction from p×q to q×q (Figure 2). Consequently, the computational cost of the proposed method is primarily affected by the column size of the observable matrix. In other words, the number of the observational basis functions determines the computational cost of the proposed approach. Usually, the size of the observable space is far smaller than the number of samples for large‐data problems. Therefore, the proposed approach can handle large‐scale data, with computational cost independent of the sample size, as the information in the data is condensed into the observable space through $B^{T}B$. Additionally, computing the null space is numerically unstable in the context of nonlinear dynamics. However, the orthogonality and ordered singular values provided by SVD enable a stable and reliable computation of the null space.

The right singular vector ξ encodes the relationship Bξ=0, which corresponds to the implicit form of the rational dynamics equation:

(10) $${\dot{x}}_{k}D\left(x\right)-N\left(x\right)=0$$

The components of ***ξ*** represent the coefficients of the numerator *N*(*x*) and denominator *D*(*x*) in the reconstructed equation. The final equation is obtained by explicitly writing *N*(*x*) and *D*(*x*) based on the grouped and thresholded components of ξ. For example, if ξ contains coefficients for terms 1, *x*, $\dot{x}$ and $x\dot{x}$, the reconstructed equation might take the form:

(11) $$\xi_{1}+\xi_{2}x+\xi_{3}\dot{x}+\xi_{4}x\dot{x}=0\to \dot{x}=\frac{\xi_{1}+\xi_{2}x}{\xi_{3}+\xi_{4}x}$$

To further refine the null space, the sparse form ξ of the null space is obtained by setting a threshold *λ*, below which elements in the null space are eliminated. The selection of *λ* directly influences the trade‐off between sparsity and fitting accuracy: A larger *λ* yields sparser models but may discard weakly contributing yet biologically relevant terms, whereas a smaller *λ* retains more terms at the risk of overfitting.

In this work, *λ* is set to a fixed small value, $1\times 10^{-7}$, based on two considerations. First, this value is comparable to the machine‐precision level relative to the magnitude of nonzero singular values in the null space, ensuring that numerical truncation does not remove dynamically significant terms. Second, prior studies on sparse identification of rational dynamics have shown that a fixed threshold in the null space, rather than a cross‐validated penalty parameter, suffices to recover parsimonious models when the observational library is appropriately constructed and the data are of sufficient quality [26, 36].

Unlike traditional SINDy approaches that require extensive tuning of a sparsity‐promoting parameter (e.g., via sequential thresholded least squares or LASSO), the role of *λ* here is purely to eliminate near‐zero coefficients arising from numerical noise, not to actively select among competing terms. Consequently, the results are robust to small variations in *λ* (e.g., within half an order of magnitude), and the fixed‐threshold strategy maintains consistency across different biological systems without introducing additional hyperparameter tuning.

### 2.3 Performance Evaluation Metrics

To comprehensively evaluate the performance of the proposed method, two complementary metrics are employed. The first is the L_0_‐based structural error, which measures the accuracy of the discovered model structure by comparing the support of the identified model with the true model structure. Let *S*_true_ represent the set of nonzero terms in the true model, and let *S*_identified_ represent the set of nonzero terms in the identified model. The L_0_‐based structural error is defined as follows:

(12) $$e_{\text{structure}}=1-\frac{\left|S_{\text{true}}\cap S_{\text{identified}}\right|}{\left|S_{\text{true}}\right|+\left|S_{\text{identified}}\backslash S_{\text{true}}\right|},$$

where $S_{\text{true}}\cap S_{\text{identified}}$ represents correctly identified terms, and $S_{\text{identified}}\backslash S_{\text{true}}$ represents spurious terms (incorrectly identified nonzero terms). This metric ranges from 0 (perfect structural match) to 1 (complete mismatch) and provides a robust measure of how well the method recovers the true model structure. This metric is closely related to the Jaccard similarity coefficient and aligns with the support recovery error in sparse regression, adapted specifically for dynamical system identification to evaluate the accuracy of identifying active terms.

The second metric is the L_2_‐based parameter error, which measures the accuracy of the estimated parameters relative to their true values. It is defined as follows:

(13) $$e_{\text{parameter}}=\frac{{\left\|\xi_{\text{true}}-\xi_{\text{estimated}}\right\|}_{2}}{{\left\|\xi_{\text{true}}\right\|}_{2}},$$

where $\xi_{\text{true}}$ and $\xi_{\text{estimated}}$ are the true and estimated parameter vectors, respectively. The norm ${\left\|.\right\|}_{2}$ denotes the Euclidean norm.

The dual‐metric approach ensures a comprehensive evaluation of both structural and parametric accuracy. The L_0_‐based structural error is particularly useful in noisy scenarios, as it explicitly evaluates the correctness of the identified model structure, whereas the L_2_‐based parameter error assesses the accuracy of the estimated parameters.

## 3 Examples and Results Analyses

In this section, we present the application of our methodology to a set of challenging biological systems, each of which was chosen for its increasing complexity and relevance to our study. In these studies, a matrix norm‐based error metric was employed to quantify the discrepancy between the estimated and actual parameters within our models is employed. Specifically, the relative error, denoted as $e_{\xi }$, is defined by the following formula:

(14) $$e_{\xi }=\frac{\|\hat{\xi }-\xi^{*}{\|}_{F}}{\|\xi^{*}{\|}_{F}}$$

where $\hat{\xi }$ represents the parameter matrix estimated by our model, and $\xi^{*}$ denotes the true parameter matrix. The norm ${∥\cdot ∥}_{F}$ refers to the Frobenius norm, a measure that assesses the square root of the sum of the squares of the matrix elements. The relative errors achieved by the proposed method for each model are listed in Table 1.

**TABLE 1:** Comparison of time cost and the relative error.

Biological network	Method	Time cost (s)	Structural error	Relative error
Michaelis–Menten kinetics	Proposed method	0.000316	0	3.46E‐15
	Implicit‐SINDy	0.004169	0	5.43E‐05
	SINDy‐PI	0.025848	0	8.19E‐15
*Bacillus* *subtilis* competence	Proposed method	0.011171	0	4.56E‐12
	Implicit‐SINDy	0.179343	0	5.24E‐08
	SINDy‐PI	0.327507	0	7.92E‐13
Penicillin production	Proposed method	0.002368	0	1.56E‐09
	Implicit‐SINDy	5.326406	0	1.89E‐08
	SINDy‐PI	—	—	—
Yeast glycolysis	Proposed method	73.910405	0	2.39E‐07
	Implicit‐SINDy	2536.343636	0	6.28E‐04
	SINDy‐PI	351.943478	0	1.54E‐07

### 3.1 Michaelis–Menten Kinetics

**FIGURE 3:**
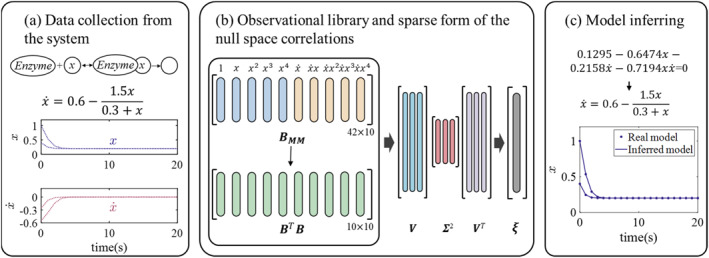
Test on the Michaelis–Menten kinetics.

The Michaelis–Menten equation, a cornerstone of enzyme kinetics for a century, describes the rate of enzyme‐catalysed reactions [37]. This model is based on a system of rational differential equations representing enzyme–substrate interactions, which can be simplified to the familiar Michaelis–Menten form under quasi‐steady‐state conditions [38]. The proposed algorithm accurately identifies the appropriate functional form from a range of potential functions and precisely extracts the correct coefficients, relying solely on time‐series data from experiments conducted with two different initial substrate concentrations (Figure 3). Initially, data were derived from the Michaelis–Menten equation.

(15) $${\dot{x}}_{k}=j_{x}-\frac{V_{\max}x}{K_{m}+x}$$

where $\dot{x}$ represents the rate of change of the variable x (substrate concentration) over time. The term $j_{x}$ denotes the generation or consumption rate of the variable x, which accounts for processes that influence the substrate concentration independently of enzymatic reactions. $V_{\max}$ is the maximum rate of the enzymatic reaction, representing the theoretical upper limit of the reaction velocity when the enzyme is saturated with the substrate. $K_{m}$ is the Michaelis constant, which indicates the substrate concentration at which the reaction rate is half of its maximum. This constant serves as a measure of the enzyme affinity for the substrate.

The model parameters were set as follows: $j_{x}=0.6$, $V_{\max}=1.5$ and $K_{m}=0.3$. Figure 3a shows the dynamic behaviour of the Michaelis–Menten system. Time‐series data were corrected from the system over a time interval from *t* = 0 to *t* = 20 s, with state variables recorded at one‐second intervals. Figure 3b shows the constructed library $B_{MM}$ that comprises 10 basis functions and it is further reduced to an observable matrix $B^{T}B$ of the size equal to the number of basis functions.

(16) $$B_{MM}=\left[1\;x\;x^{2}\;x^{3}\;x^{4}\;\dot{x}\;x\dot{x}\;x^{2}\dot{x}\;x^{3}\dot{x}\;x^{4}\dot{x}\right]$$

Then, SVD analysis is then applied to the reduced observable matrix to obtain the null space. Figure 3c first shows the four identified active terms and the corresponding coefficients. They are further rearranged to get the final inferred model, which is equivalent to the original one, exhibiting an exceptionally small relative error of 3.46 × 10^−15^.

### 3.2 *Bacillus subtilis* Competence

After exploring the Michaelis–Menten kinetic model, we shifted our focus to another regulatory model with two state variables: the competence in *Bacillus subtilis*. Helge et al. [39] regulated the expression dynamics of genes in *Bacillus subtilis* by employing genetic code expansion technology, thereby influencing cellular behaviour and metabolic processes.

In *Bacillus subtilis*, the development of competence involves a complex gene regulatory network. The ComK protein serves as the primary regulator of competence development and is subject to precise control of its expression. A simplified kinetic model that captures the dynamic changes in the concentrations of ComKand ComS proteins can be described by the following system of equations [40]:

(17) $${\dot{x}}_{1}=a_{1}+\frac{a_{1}x_{1}^{2}}{a_{3}+x_{1}^{2}}-\frac{x_{1}}{1+x_{1}+x_{2}}$$

(18) $${\dot{x}}_{2}=\frac{b_{1}}{1+b_{2}x_{1}^{5}}-\frac{x_{2}}{1+x_{1}+x_{2}}$$

In the first equation, $a_{1}$ represents the basal generation rate. The second term describes how the ComK protein activates its production through a positive feedback loop, with the Hill function illustrating the cooperative action of ComK protein within the regulatory complex. The third term accounts for the degradation of ComK mediated by a third unmeasured protein, MecA. In the second equation, the first term represents the negative feedback loop of ComS protein production mediated by ComK, which helps to maintain the homoeostasis of protein concentrations within the cell. The second term again reflects the degradation of ComS mediated by MecA.

**FIGURE 4:**
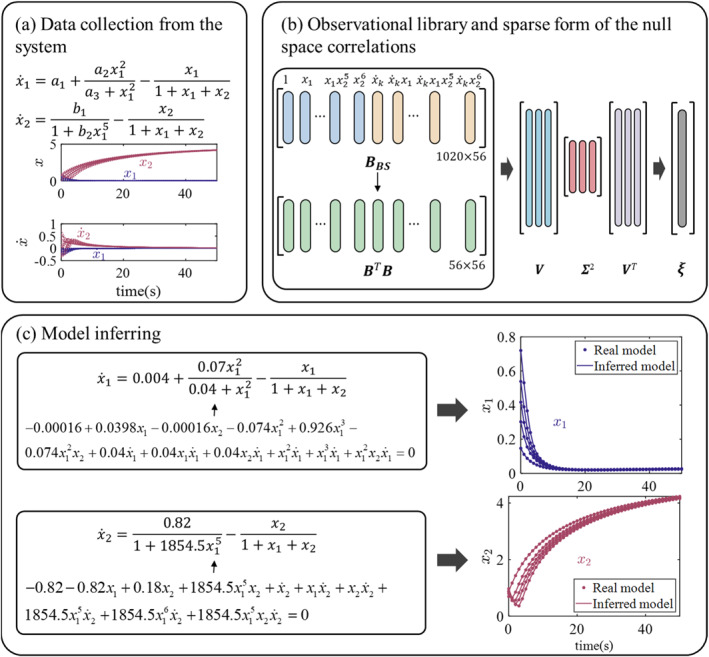
Test on the *Bacillus subtilis* competence system.

The parameters were configured as $a_{1}=0.004$, $a_{2}=0.07$, $a_{3}=0.04$, $b_{1}=0.82$ and $b_{2}=1854.5$. Figure 4a shows the dynamic behaviour of the system with two state variables. Time‐series data were generated from this system over a time interval of *t* = 0 to *t* = 50 s, with state variables recorded at one‐second intervals. Two state variables, each initialised with 20 random values, generate a total of 20 time series. In constructing the kinetic model of competence in *Bacillus subtilis*, the challenge of this model is to accurately identify the dynamic interdependencies of two state variables accurately and to handle the polynomial functions in the denominator of the equations. Figure 4b illustrates the construction of the observable matrix, which is derived from a library of basis functions. SVD analysis is then applied to the reduced observable matrix to extract the null space.

**FIGURE 5:**
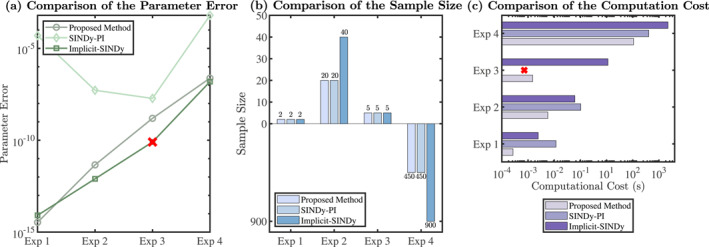
Performance comparison between the proposed method and the implicit‐SINDy.

Figure 4c shows the identified 12 active terms for $x_{1}$ and 10 for $x_{2}$, along with their corresponding coefficients. These active terms and coefficients were rearranged to construct the final inferred model, which closely matched the behaviour of the original system. Similar to the first example, the identified model parameters are almost identical to the original parameters (see Table 2), with a relative error of 5.39 × 10^−9^. The number of samples required is half of the 40 samples needed by implicit‐SINDy, achieving a time reduction of over 95% (see Table 1 and Figure 5).

**TABLE 2:** Parameter identification for *Bacillus subtilis* competence.

Parameter	Units	True value	Extracted value
*a*_1_	[nM/s]	0.004	0.00400000000000467
*a*_2_	[nM/s]	0.07	0.0699999999999961
*a*_3_	[nM]	0.04	0.0400000000000038
*b*_1_	[nM/s]	0.82	0.819999990065821
*b*_2_	[nM]	1854.5	1854.14000669824

### 3.3 Penicillin Production

Penicillin, a renowned antibiotic discovered by Alexander Fleming in 1928, has revolutionised the field of medicine by providing an effective treatment against bacterial infections [41]. It is a secondary metabolite produced by the fungus Penicillium and has been extensively studied for its mode of action, which primarily involves the inhibition of bacterial cell wall synthesis [42]. The production of penicillin has been a subject of significant industrial and academic interest due to its therapeutic importance, leading to the development of various fermentation processes to optimise yield [43, 44].

The production of penicillin can be described by a nongrowth‐associated product formation kinetics, which takes into account the specific penicillin production rate and the influence of substrate inhibition. The equation that encapsulates the dynamics of penicillin production is as follows:

(19) $${\dot{x}}_{1}=\mu_{p}\frac{X_{2}}{K_{p}+x_{2}\left(1+\frac{x_{2}}{K_{1}}\right)}\frac{x_{3}^{3}}{K_{op}x_{4}+x_{3}^{3}}x_{4}-{Kx}_{1}-x_{1}\frac{F}{x_{5}}$$

where the state variables represent the penicillin concentration (*x*_1_), the substrate concentration (*x*_2_), the dissolved oxygen concentration (*x*_3_), the biomass concentration (*x*_4_) and the volume (*x*_5_).

Considering the simulation without oxygen limitation, Equation (15) reduces to a form that does not include the oxygen limitation with *K*_op_ = 0,

(20) $${\dot{x}}_{1}=\mu_{p}\frac{X_{2}}{K_{p}+x_{2}\left(1+\frac{x_{2}}{K_{1}}\right)}-{Kx}_{1}-x_{1}\frac{F}{x_{5}}$$

**FIGURE 6:**
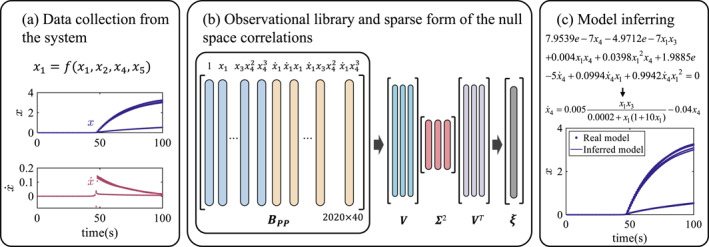
Test on the penicillin production kinetics.

Bajpai and Reuss [45] provided a simulation platform for conducting a comprehensive simulation of penicillin production. Figure 6a shows the dynamic behaviour of the penicillin production system. Time‐series data were generated from this platform, with a total runtime of 100 h and hourly sampling to capture system dynamics. The true parameter values were determined the same as in ref. [45] (see Table 3). The time‐series dataset was generated by initialising the penicillin concentration with 20 initial conditions.

**TABLE 3:** Parameter identification for penicillin production.

Parameter	Units	True value	Extracted value
*K*	[h]	0.04	0.004999999986990
*K*_p_	[g/L]	0.0002	2.000002043237612 × 10^−4^
*μ*_p_	[h]	0.005	0.039999999932917
*F*	[1/h]	0	0

Figure 6b illustrates the process of building an observable matrix. SVD analysis is then performed on the reduced observable matrix to select 7 active terms from a pool of 40 candidates. Figure 6c shows the reconstructed model, which exhibits a strong consistency with the original system. The extracted parameters exhibit a relative error of only 8.89 × 10^−10^ from the true values. Across 50 experimental runs, the approach demonstrates an average runtime of 0.00133 s, highlighting its computational efficiency. These results indicate that the proposed method achieves high accuracy and low computational cost when applied to the penicillin production equation.

### 3.4 Yeast Glycolysis

Yeast glycolysis is a metabolic pathway involving a sequence of enzyme‐catalysed reactions that break down glucose into two molecules of pyruvate within yeast cells, generating ATP and NADH, which provide energy and reduce power for the cell. This process involves multiple key enzymes and regulatory steps, making its dynamic characteristics and regulatory mechanisms a focal point of research in metabolic engineering and systems biology.

**FIGURE 7:**
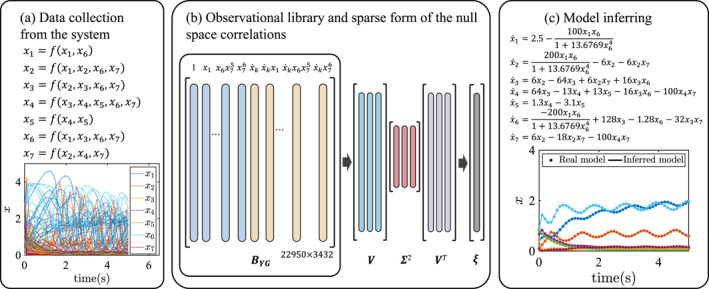
Test on the yeast glycolytic metabolic network.

In biochemical kinetics, the oscillating model of yeast glycolysis [46] has become a benchmark case for verifying the accuracy of inference methods [47]. This model was employed here to assess the performance of the proposed model identification method (Figure 7). It involves the concentration dynamics of seven key biochemical species, described by a set of ordinary differential equations (ODEs).

(21) $${\dot{x}}_{1}=c_{1}+\frac{c_{2}x_{1}x_{6}}{1+c_{3}x_{6}^{4}}$$

(22) $${\dot{x}}_{2}=\frac{d_{1}x_{1}x_{6}}{1+d_{2}x_{6}^{4}}+d_{3}x_{2}-d_{4}x_{2}x_{7}$$

(23) $${\dot{x}}_{3}=e_{1}x_{2}+e_{2}x_{3}+e_{3}x_{2}x_{7}+e_{4}x_{3}x_{6}$$

(24) $${\dot{x}}_{4}=f_{1}x_{3}+f_{2}x_{4}+f_{3}x_{5}+f_{4}x_{3}x_{6}+f_{5}x_{4}x_{7}$$

(25) $${\dot{x}}_{5}=g_{1}x_{4}+g_{2}x_{5}$$

(26) $${\dot{x}}_{6}=\frac{h_{1}x_{1}x_{6}}{1+h_{2}x_{6}^{4}}+h_{3}x_{3}+h_{5}x_{6}+h_{4}x_{3}x_{7}$$

(27) $${\dot{x}}_{7}=j_{1}x_{2}+j_{2}x_{2}x_{7}+j_{3}x_{4}x_{7}$$

The state variables are defined as follows: the concentration of glucose (*x*_1_), the pool of triose phosphates, glyceraldehyde 3‐phosphate, and dihydroxyacetone phosphate (*x*_2_), 1,3‐bisphosphoglycerate (*x*_3_), the pool of pyruvate and acetaldehyde (*x*_4_), NADH (*x*_5_), ATP (*x*_6_) and the concentration of the coupling substance in the external solution (*x*_7_).

The true parameter values and simulation conditions for glycolysis are consistent with those specified in ref. [31]. Figure 7a shows the visualisation of the state variables of the yeast glycolytic metabolic network. A total of 450 time‐series data are collected from this system over a time interval of *t* = 0 to *t* = 5 s, with state variables sampled at intervals of 0.1 s. Figure 7b shows the process of building an observable matrix using a sixth‐degree library of 3432 basis functions and SVD analysis on the observable space to obtain the sparse form of null space. Subsequently, all active terms from the library and the corresponding coefficients are extracted from the null space.

Table 4 presents the true coefficient values for the model, alongside the coefficient extracted using the method of this study. These results demonstrate that the proposed method performs exceptionally well in the glycolysis model. Additionally, the time efficiency of the proposed method compared to implicit‐SINDy is particularly pronounced, especially for the equations involving *x*_1_, *x*_2_ and *x*_6_. The sample size was reduced by half, resulting in a time savings of over 95% (see Table 1, Table 5 and Figure 5).

**TABLE 4:** Parameter identification for yeast glycolysis.

Parameter	Units	True value	Extracted value
*c*_1_	[mM/min]	2.5	2.4999999999592
*c*_2_	[1/(mM min)]	−100	−100.000000000628
*c*_3_	[mM^−1/4^]	13.6769	13.6768670565533
*d*_1_	[1/(mM min)]	200	200.000000044651
*d*_2_	[mM^−1/4^]	13.6769	13.6768670615051
*d*_3_	[1/min]	−6	5.9999998238964
d_4_	[1/(mM min)]	−6	6.00000264943159
*e*_1_	[1/min]	6	5.99999999999921
*e*_2_	[1/min]	−64	63.9999999999997
*e*_3_	[1/(mM min)]	6	6.00000000000058
*e*_4_	[1/(mM min)]	16	15.9999999999994
*f*_1_	[1/min]	64	63.9999999999833
*f*_2_	[1/min]	−13	13.0000000000165
*f*_3_	[1/min]	13	13.0000000000096
*f*_4_	[1/(mM min)]	−16	15.9999999999938
*f*_5_	[1/(mM min)]	−100	99.9999999999579
*g*_1_	[1/min]	1.3	1.3
*g*_2_	[1/min]	−3.1	−3.1
*h*_1_	[1/(mM min)]	−200	200.000147936965
*h*_2_	[mM^−1/4^]	13.6769	13.6768756830724
*h*_3_	[1/min]	128	128.000766936781
*h*_4_	[1/(mM min)]	−32	32.0007682737036
*h*_5_	[1/min]	−1.28	1.28000471062441
*j*_1_	[1/min]	6	5.99999999999971
*j*_2_	[1/(mM min)]	−18	17.9999999999998
*j*_3_	[1/(mM min)]	−100	−100.000000000011

**TABLE 5:** Main features of studied models.

Proposed method	State variables	Degree of polynomial	Size of library	Data sample size
Michaelis–Menten kinetics	1	4	10	2
*Bacillus* *subtilis* competence	2	6	56	20
Yeast glycolysis	7	6	3432	450

Implicit‐SINDy	State variables	Degree of polynomial	Size of library	Time series measurements
Michaelis–Menten kinetics	1	4	10	2
*Bacillus* *subtilis* competence	2	6	56	40
Yeast glycolysis	7	6	3432	900 (^a^2250)

^a^ Implicit‐SINDy requires more data to determine Equation (16).

In Figure 5, Exp 1, Exp 2, Exp 3 and Exp 4 represent the Michaelis–Menten kinetics, the regulatory network for competence in *Bacillus subtilis*, the penicillin production kinetics and the yeast glycolytic metabolic network, respectively. Figure 5a shows the proposed method achieves lower relative errors than the implicit‐SINDy for all four experiments. Figure 5b shows the proposed method requires fewer samples than the implicit‐SINDy, where a notable difference in Exp 2 and Exp 4 can be observed. Further, in Figure 5c, it is shown that the proposed method has a lower computational cost than the implicit‐SINDy in all experiments.

### 3.5 Noise Experiment

In the field of nonlinear dynamics, one of the primary challenges in extracting equations from data is dealing with low‐quality or noisy data [48]. The proposed method can recover rational dynamic equations in the presence of noise. Hence, compared to implicit‐SINDy, the proposed method can identify a most possible model structure without relying on a threshold value with the assumption that only one model exists in the studied problem [36]. Under large noise situation, the singular values could all be large, but under the prior assumption that there is only one model to be identified from the data, the right singular vector that corresponds to the smallest singular value can be always chosen as the identified model structure.

The performance of the proposed method under noisy conditions is evaluated by comparing it with implicit‐SINDy and SINDy‐PI, on the Michaelis–Menten kinetics. Gaussian noise is added to the state measurements, with noise levels ranging from 10 to 7 to 0.5 (23 distinct levels). Given the significant impact of noise on parameter precision, the primary focus is on structural accuracy. Derivatives of noisy data are calculated using total‐variation regularised difference (TVRegDiff) [49] derivatives, which requires hyperparameter tuning and may introduce derivative aliasing. Consequently, the first 30% and the last 30% of the time series data generated for each initial condition are truncated.

The candidate library consists of 10 terms, with 4 active terms expected in the correct model structure. A total of 2400 distinct initial conditions with magnitudes ranging from 0 to 12.5 are generated randomly. These initial conditions are simulated using the ode45 solver, with a time step of *dt* = 0.1 and a time horizon of *T* = 5. To quantify the structural accuracy of the identified model, a benchmark value of 0.25 is established for structural error. A value below 0.25 indicates good identification of the correct model structure, where a value of 0.25 represents the identification of three correct terms (missing one term), a value of 0.2 indicates the identification of all four correct terms plus one additional incorrect term and a value of 0 represents perfect structural accuracy. Values above 0.25 signify poor model structure identification.

**FIGURE 8:**
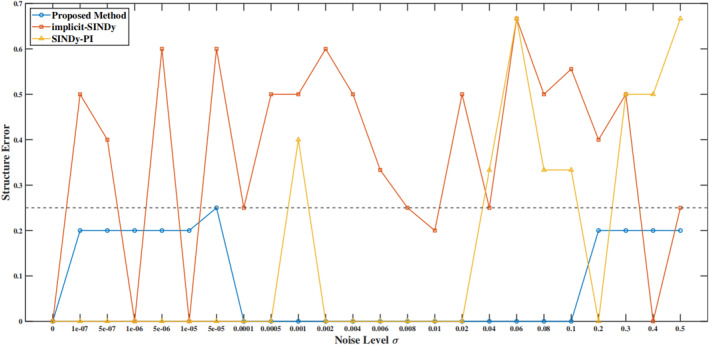
Relative error at different noise magnitudes.

Figure 8 illustrates the structural errors of the three methods on the Michaelis–Menten kinetics under different noise levels. The proposed method demonstrates robust performance across all noise levels, consistently achieving structural error values below 0.25, even at high noise levels (*σ* ≥ 0.1). In contrast, SINDy‐PI performs well at low to moderate noise levels but fails at high noise (*σ* ≥ 0.1). Implicit‐SINDy shows poor performance even at low noise levels (*σ* < 0.0001), with structural errors consistently above 0.25, indicating significant challenges in model recovery. Overall, the proposed method performs well across all noise levels, with slightly inferior performance compared to SINDy‐PI at low noise but superior overall robustness.

## 4 Discussion and Conclusion

To infer the nonlinear dynamics of rational biological network models, the method proposed in this study identifies correlations in a set of basis functions using the null space solution of a data observable matrix constructed by these basis functions. The basis functions are the mixed state and derivative terms of a rational function. This approach does not require iterative optimisation relying on sparsity constraints and offers a dimensionality‐reduced observable matrix for rational model identification. Furthermore, the introduction of a small threshold helps promote the sparsity of the null space.

A core advantage of this method is its ability to extract a set of orthogonal bases from the observable matrix, which represents the dominant modes of system state changes. Compared to traditional parameter inference methods, the proposed method significantly reduces the computational resource demands, particularly when dealing with large‐scale datasets, which is crucial for addressing big data challenges in modern bioinformatics.

However, the choice of the observational library directly influences the model accuracy. In addition, model construction and parameter inference may still encounter difficulties when dealing with highly complex nonlinear systems. Future research should focus on optimising the construction of the basis function library and developing more efficient algorithms to accommodate a broader range of biological network models.

In conclusion, this study provides a novel perspective on the parameter inference for rational form biological network models. By effectively handling large‐scale data and complex models, it exhibits itself as a useful tool for advancing the understanding of biological system dynamics.

## Author Contributions

**Hongtao Zhu:** conceptualization, methodology, resources, validation, visualization, writing – review and editing. **Longwei Zuo:** conceptualization, data curation, formal analysis, methodology, writing – original draft. **Chunjian Pan:** conceptualization, formal analysis, methodology, resources, supervision, validation, writing – review and editing. **Qingchao Jiang:** conceptualization, formal analysis, investigation, methodology, project administration, supervision, writing – review and editing. **Zhixing Cao:** investigation, project administration, supervision, writing – review and editing.

## Funding

The authors gratefully acknowledge the support from the following foundations: National Natural Science Foundation of China under Grant 62322309 and Shanghai Science and Technology Innovation Action Plan under Grant 23S41900500.

## Conflicts of Interest

The authors declare no conflicts of interest.

## Data Availability

The data that support the findings of this study are available from the corresponding author upon reasonable request.
